# Medico-Chirurgical Transactions, Vol. 27

**Published:** 1845-04

**Authors:** 


MEDICO-CHIRURGICAL TRANSACTIONS, PUBLISHED BY THE ROYAL MEDICAL AND CHIRURGICAL SOCIETY OF LONDON. Volume the Twenty-seventh, (the Ninth of the Second Series), 1844. Continued from page 32.

### XVI. ON OBSTRUCTIONS OF THE BRANCHES OF THE PULMONARY ARTERY. By *James Paget.*

THE obstructions treated of in this paper, are those produced by the coagulation of blood during life in the branches of the pulmonary artery. They are not of rare occurrence, and, the author observes, are always important, for they are sometimes the sole or chief cause of death, and must in all cases seriously affect the progress of the diseases with which they are associated. Yet they have hitherto scarcely attracted attention, and in the only paper which he can find expressly treating of them (one by M. C. Baron, in the Archives Générales de Médecine, Paris 1838) there are but four cases related, and these give a very imperfect account of the nature of the disease and of the circumstances in which it occurs.

"From the arrangement of the pulmonary arteries, between which there is no anastomosis, except in their capillaries and smallest branches, it results that whenever the flow of blood through the capillaries of any part of a lung is prevented, there must also be a stagnation of the blood in all the branches from which those capillaries are derived; and in these circumstances, the blood coagulates in the vessels, and passes through various changes." 163.

Now these conditions are present in several diseases;—First, in *pulmonary apoplexy*, especially in that form of it in which the blood collects in a defined and compact dark mass. In all, or in a great majority of these cases, the branches of the pulmonary artery leading to the seat of effusion are blocked up by coagula, which present the distinguishing characters of those formed long previous to death.

Secondly.—The capillary circulation is usually obstructed or much hindered in the advanced stages of pneumonia, and the arrested blood coagulates in those branches which correspond to the inflamed and consolidated portions of lung. So far as the author has seen, "the coagula in these cases are not sufficiently large to fill the vessels; circulation enough, therefore, may go on to prevent gangrene; but still, such permanent, though imperfect obstacles to the passage of blood must materially impede and endanger the recovery of the diseased part, and no doubt the formation of these clots often precedes the complete obliteration of the pulmonary arteries, when, after partial recovery from pneumonia, the diseased portion of the lung has healed and contracted. Sometimes, indeed, coagula thus formed in pneumonia are fatal. They were so in one of the cases mentioned by M. Baron. A patient of M. Louis' was suddenly seized with symptoms of asphyxia during convalescence from pneumonia: he died in five or six hours, and 'nothing could be found to explain his death, except some soft non-adherent clots, which obstructed the cavity of the pulmonary artery.'"

Thirdly.—When the matter of medullary cancer or of softened scirrhus passes into the blood, and, circulating with it, is stopped in the lungs, the branches of the pulmonary artery may be to a great extent filled by it and by coagulated blood or fibrine mixed with it. Mr. Paget relates a case which affords a good example of this fact. It was the case of a woman affected with carcinoma of the liver. On examination of the body, it appeared that the cancerous substance had been conveyed with the blood from the liver to the lungs, where, being arrested and obstructing the minute vessels, it had permitted fresh substance with blood to accumulate behind it. Some other cases in which similar appearances were found in the lungs are also mentioned.

Fourthly.—The branches of the pulmonary artery are often blocked up by clots formed during life in those who die with great œdema of the lungs. In some of these cases the coagulation is probably consequent, as in pulmonary apoplexy from disease of the left side of the heart, on the obstruction to the passage of the blood through the capillaries. But the author is of opinion, that in general some further condition is necessary, and he gives two cases of coagula occurring with œdema of the lungs, in one of which sufficient evidence of a morbid state of the blood is afforded by the signs of its early decomposition after death.

Mr. Paget remarks that, in these four classes of cases, " the coagulation of the blood in the pulmonary arteries may be regarded as a secondary phenomenon; for it usually appears as the consequence, either chiefly or entirely, of the obstruction in the capillaries. Whenever that obstruction is complete, and prevails through the whole of both lungs, death by asphyxia must ensue before the arrested and coagulated blood can undergo any structural changes; and such changes can take place only when the capillary circulation can be carried on in some parts of the lungs, at the same time that in others the blood is stagnant."

The author believes, also, that in some other diseases under the same essential conditions similar coagula are often formed. But there are other classes of cases in which their formation appears to be a primary disease, or in which at least it cannot be regarded as the result of mere obstruction in the capillaries. Of these he has observed three examples. The first is the case of a woman, 29 years of age, who was admitted into St. Bartholomew's Hospital, May 5th, 1843, with symptoms of rheumatism, attended with a good deal of depression of the system, and a rapid pulse and respiration. After a few days the abdomen became distended, and rather painful on pressure; on the next day she complained of great tenderness in the right iliac region; and on this day also (May 9th) the præcordial region was found tender, and on auscultation, a distant bellows sound was heard at the base of the heart accompanying the systole.

" On the 10th, the cheeks and forehead were covered with an erysipelatous blush, and numerous red acuminated papulæ had appeared upon the chest, the urine was very irritating, and smelt offensively, sloughs has begun to form upon the sacrum, and the weakness and depression were increased. On the morning of the 11th, after passing a comfortable night, she was suddenly seized with a sensation of great tightness in the præcordial region, violent palpitation of the heart, and the most urgent dyspnœa. The attack lasted for an hour, and then she returned to nearly the same state as she had been in before it; but from this time she sank more rapidly. Upon auscultation, no respiratory murmur was heard below the right breast, and it was dull on percussion; the habitual dyspnœa became greater, the sloughs on the nates and sacrum extended, the abdomen became again tympanitic and tender, especially about the right iliac region, she vomited several times, and died in the afternoon of the 13th." 176.

On examination 40 hours after death, all the blood which appeared in the ordinary course of the dissection was found either fluid or coagulated in soft black masses. The pericardium contained half an ounce of fluid, and there was a few slender adhesions between the trunks of the larger vessels. No particular disease of the heart and principal vessels. A small quantity of fluid in each pleural sac. Lower lobes of both lungs œdematous and gorged with blood. Trachea and bronchi healthy. "Nearly half the branches of the pulmonary artery, from those of the second order, to those of the fifth and sixth (and probably to yet smaller branches), were blocked up by old coagula of blood. These were cylindrical, soft and grumous, and in colour were a mixture of pale pink and dirty greyish white, with spots and blotches of deep crimson. They were not more numerous in one lung than in the other, and were irregularly scattered through all parts of each. They did not quite fill the vessels which contained them, but at various parts they adhered closely to the walls. The trunk of the pulmonary artery, and many of the branches which did not contain coagula of the kind just described, contained fluid and softly clotted black blood." Abdominal and pelvic organs healthy.

In the next case, a woman, 70 years of age, who had suffered for five weeks from a bad cold, was taken into the hospital in a declining state. On the day after her admission, she seemed to improve a little, but next day she sank rapidly, and Dr. Burrows "remarked an extreme hurry of the circulation, with feebleness of the pulse and great prostration of strength, very similar to those observed in the preceding case." On examination of the body 36 hours after death, there was no other particular disease except a small patch of compact pulmonary apoplexy with some diffused apoplexy around it, at the anterior and lower border of the left lung; and "in each lung, one of the superior and one of the inferior main branches of the pulmonary artery were blocked up by a large, firm, mottled, clot of blood, which, from itself as a trunk, sent branches into two or more of the next order of branches of the artery. The colours of the clots were black, deep-crimson, rusty, pink and yellowish, in various irregularly-mingled shades; they were moderately firm, of nearly uniform consistence throughout, and capable of being rubbed into a thick grumous substance; they adhered so firmly by parts of their surfaces to the adjacent walls of the vessels, that they could not be smoothly removed. The branches of the largest clots did not extend far into the arteries, but terminated abruptly in the arterial ramifications immediately proceeding from those in which they lay. But beyond their terminations, many smaller branches of the pulmonary arteries in all parts of the lungs contained short, firm, dry, mottled, and adherent clots, variously coloured. None of these was long or much ramified; none was continued through more than two branches; and many of them were not more than half or three-quarters of an inch long; some were even shorter, and lay like bits of larger clots upon the walls of the vessels. They had all the same characters as those already described; and the portions of lung adjacent to and beyond them, were not different from the rest.

"Moreover, in several of the larger branches of the pulmonary artery, there were appearances of clots of blood formed like those just described, and having been still further altered and organized. These were pale, semi-transparent, soft, and flattened narrow bands, attached firmly to the walls of the artery, and presenting all the characters of the organized clots which I have sometimes seen adhering to the walls of divided arteries. They were from one quarter of an inch to an inch in length, and about one-tenth of an inch wide; a few were fixed, in their whole length, to the walls of the artery, but most of them by their ends only, so that a probe could be passed under them. Among the clots there were all gradations, between those last and those first described; and in one instance, one of the largest of the more recent clots was continuous, with a flat, semi-transparent, and adherent portion, like those which had existed for the longest time.

"The branches of the pulmonary arteries which were thus obstructed, were smooth and polished internally, but in many places had fine, scattered, and grouped grains of yellow deposit in their coats. They were all of natural size; and the pulmonary veins were healthy."

The cavities of the heart were dilated and hypertrophied, and contained adherent coagula. The mitral and aortic valves were thickened, but able to discharge their functions. Numerous yellow deposits in the coats of the aorta. The large veins and the systemic arteries, as far as they were examined, were healthy.

The third case, was that of a young girl who had long lived in the miseries of poverty and prostitution, admitted into the hospital for gonorrhœa, pale, haggard and scarcely able to walk. After a month, signs of pleuro-pneumonia came on and she soon died. We have not space for the minute particulars given of the post-mortem examination.* It is sufficient to remark that, in the lower lobes of both lungs, all the pulmonary arteries from the origin of the main branch to the most distant branches were full of old fibrinous coagula, and that there were also about twenty firm and compact masses of *pulmonary apoplexy* of very irregular form. The clots in this last case were distinguished by being laminated, as if formed by the deposition of successive layers from the wall towards the axis of the artery. In all the others, the particles of the clot were irregularly arranged: they were throughout various in both colour and consistence. Some were partially softened; indicating that the blood had died before the general death of the body; others, in which the blood had, probably, become further organized, had acquired firm adhesions to the walls of the vessels; and in the last case but one, it was evident that the clots were fully organized, and had acquired organic connexions with the adjacent parts.

"In this respect the case is peculiarly interesting, for it proves that blood, though coagulated in circumstances of disease, is yet capable of further organi-

---

* In this case there were only two valves in the pulmonary artery: they were both diseased.

zation, when placed in conditions compatible with the maintenance of its life; and further, that the coagulation of the blood in the large pulmonary arteries, is not necessarily either the cause or the attendant of fatal disease; for several weeks must have been occupied in effecting the complete organization to which these clots had attained." 187.

Mr. Paget's character as an accurate observer, and the little attention hitherto bestowed on ante-mortem clots in the branches of the pulmonary artery, have led us to quote rather largely from this paper, though we do not see that any important practical inferences can be drawn from it, and the author admits his inability to connect the symptoms presented by the three patients whose cases we have given with the morbid appearances. It appears to us that, in the two latter at least of these cases, the blood had from some cause become morbidly altered, and rendered unfit for circulation; and, as a natural consequence, that obstruction had taken place in the lungs, the organs which usually suffer when the blood is contaminated by pus in phlebitis, or by the germs of cancer in malignant disease. This paper will however be useful in directing attention to the condition of the pulmonary artery in cases of fatal disease, and further investigation will show the degree of importance to be attached to the clots which form in its branches during the close of life.

### XVII. On the Composition of the Meconium, and of the Vernix Caseosa, or lubricating Matter of the New-born Infant. By *John Davy*, M.D. F.R.S.

The microscopical character of meconium is very distinctive, and well displays its compound nature: it exhibits a confused mixture of globules, plates, and molecules. The globules about $\frac{1}{3000}$th of an inch in diameter, are very abundant, and form a principal part of the whole. Judging from their form and size, their insolubility in water and alcohol, they may be inferred to consist chiefly of mucus. The plates, which are tolerably abundant, are of two kinds, One kind is of irregular form, somewhat granular, varying in size from about $\frac{1}{2000}$ to $\frac{1}{1000}$ of an inch in diameter, insoluble in water, hot or cold alcohol, the dilute acids and alkalies—like epithelium scales, which the author believes them to be. The other kind are of a regular kind, chiefly rhomboidal, of great thinness, and perfect transparency, insoluble in water, acids, and cold alcohol, but readily soluble in hot:—properties indicative of cholesterine. The molecules vary in size from $\frac{1}{8000}$ to $\frac{1}{20000}$ of an inch in diameter; and being insoluble in water, and soluble in alkaline ley, may be considered as consisting chiefly of fatty matter.

Besides these ingredients, to which the meconium owes its thick consistency and viscid nature, there is another portion from which the mass derives its colour and taste, and probably its power of resisting putrefaction, and which seems identical with the colouring and sapid matter of bile, being soluble in water and alcohol. The specific gravity of meconium exceeds that of water; it sinks in a saturated solution of common salt of the sp. gr. 1148. The quantities of meconium which the author has obtained have been too small to admit of accurate analysis; but in a specimen obtained from a healthy child immediately after birth, the proportion of ingredients was determined, and the results per cent. were about as follows :—

| | |
|---|---|
| 23·6 | mucus and epithelium scales. |
| ·7 | cholesterine and margarine. |
| 3·0 | colouring and sapid matter of bile and oleine. |
| 72·7 | water. |
| 100·0 | |

These proportions the author believes may be considered pretty correct. A portion of the same meconium was incinerated. It burnt with a bright flame, and left ·69 per cent. of reddish ash, chiefly peroxide of iron and magnesia, with a trace of phosphate of lime and common salt.

The vernix caseosa, under the microscope, is found to be composed of granules, plates, and molecules; the plates constituting the principal part. These have the properties of epithelium scales, the granules, those of fatty matter, as also the molecules. The plates are insoluble, both in weak acids and in alkaline leys, and in cold and hot alcohol; are of irregular form, varying in size from about $\frac{1}{666}$ to $\frac{1}{1000}$ of an inch in diameter, and very thin. The vernix is apparently lighter than water, on which it floats; but this is owing to the air entangled in it. Of a butteraceous consistence in its ordinary state, at a temperature of 60°, it hardens on reduction of temperature, and becomes almost semifluid when its temperature is raised to 100°,—admirably adapting it for a lubricating substance in parturition.

A single specimen of the lubricating matter, of great purity, was found to consist of—

| | |
|---|---|
| 13·25 | epithelium plates. |
| 5·75 | oleine. |
| 3·13 | margarine. |
| 77·37 | water. |
| 100·00 | |

Dr. Davy remarks that, theoretically considered as regards the origin of the two substances treated of, the preceding results seem to point out distinctly, that both are excretions, the meconium chiefly derived from the liver, and the lubricating matter from the skin. He alludes to the opinion of Raspail, that a portion of the meconium consists of intestinal villi, but observes that he has sought in vain for the appearances which Raspail describes. Vauquelin and Buniva, after examining the vernix caseosa, were led to infer that it is not an excretion from the infant, but a deposit on its surface from the liquor amnii. Bichat rejected this view merely from the circumstance that no such deposit is found on the umbilical cord, or on the inner surface of the amnios, and came to the conclusion, which seems most just, that it is derived from the skin of the fœtus, and is a secretion similar to that which takes place after birth in many parts of the cutaneous system.

XVIII. On Paracentesis Thoracis as a Curative Measure in Empyema and Inflammatory Hydrothorax. By *Hamilton Roe,* M.D. Oxon.

The author commences this paper by stating that the opinion very generally prevails amongst the most eminent members of the profession, that the operation of tapping the chest affords but little hope of curing either empyema or hydrothorax, and that therefore it should never be performed until the difficulty of breathing, caused by the pressure of the fluid, becomes so urgent as to threaten immediate death. The reasons assigned for this opinion are—" First, that the re-accumulation of fluid will commence immediately after its evacuation has been effected, and can be prevented by the action of the absorbents only ; and, therefore, that we ought to rely upon them, in the first instance, for its removal. Secondly, that the operation itself is attended with very considerable danger. And, Thirdly, that the majority of those cases in which the operation has been performed, has terminated fatally."

After mentioning that Sir A. Cooper, Laennec, Stokes, and some others, speak unfavourably of the operation, the author remarks that " no modern physician or surgeon has recommended it as a curative measure." We should infer from this statement that Dr. Roe is imperfectly acquainted with the opinions entertained on this subject by the best modern practitioners, if he had not directly contradicted himself at page 214, by observing:—" It (the operation) has been recommended by many eminent physicians and surgeons on the Continent, in America, and in our own country—viz. Baron Larrey, T. P. Frank, Bell, Dr. Elliotson, Dr. Forbes, Dr. Copland, Dr. C. J. B. Williams, Dr. E. Harrison, the late Dr. Davis, and several others." We leave it to the author to reconcile these two statements. The fact is, paracentesis thoracis is not in such " very general disrepute" as the author would *in limine* lead us to believe, as he has himself shewn in the passage just quoted. The late Dr. Davies decidedly advocated this plan of treatment; and Dr. Watson, instead of waiting in cases of empyema until suffocation was threatened, advises, " whenever the effused liquid consists of pus, it should be let out."*

We must admit, however, that the opinions of the profession respecting this operation are sufficiently unsettled to warrant Dr. Roe in an attempt to estimate its value, and to lay down rules for its application.

In order to ascertain how far the objections to the operation were derived from the results which followed its performance, he states, that he has collected all the cases of this operation which had been published in the English language, between the years 1812 and 1832 inclusive.

These cases are arranged in a tabular form, and certain particulars are stated in separate columns, to enable the Society to form a judgment of the causes of the failure or success of the operation. The table includes 39 cases: of these 28 recovered, and 11 died. Encouraged by this result, Dr. Roe tapped all cases of empyema and hydrothorax, in which the means adopted did not after a few weeks produce a sensible diminution of the effused fluid, and he found the success attendant upon this practice to

---

* Medical Gazette, Vol. I., 1841—42, p. 249.

exceed his most sanguine expectations. Before describing these cases, he makes some obervations in reply to the principal arguments brought forward against the operation. We shall notice the principal. It is said that the admission of air during the operation is attended with danger, and frequently renders it unsuccessful. The author admits that air readily enters the pleura—it occurred in all cases under his observation, but in none of them did it produce any permanently evil effect: in one instance only did it cause even temporary inconvenience. The air by its pressure on the lung produced dyspnœa, which was relieved by the air being pumped out with an ordinary syringe. Another objection which is noticed, is one advanced in a posthumous essay of the late Dr. Hope, viz. that all cases of empyema really curable, are curable without paracentesis. The author remarks:—

"This conclusion appears to have been drawn from the results of thirty-five cases of that disease, recorded in the paper just alluded to, in which the exhibition of mercury, continued until the patients had been brought completely under its influence, was followed by the removal of the fluid. Now without calling in question the existence of empyema in all these cases, or seeking for the proofs of its presence, I would ask, before I proceed to combat the objection, what is meant by the cure of empyema? If it be the simple removal of the fluid, I am not inclined to dispute the *possibility* of effecting so much by the power of mercury, for I have had many cases in which the symptoms of fluid in the chest have disappeared under its action: but this is not necessarily a cure; for the lung which has been compressed by the fluid is often left so altered in its structure, that it never again can perform its healthy function. But if it be meant, that in all curable cases of empyema, mercury, exhibited in the manner prescribed, will not only cause the absorption of the fluid, but the restoration of the lung to health, the experience I have had of the effect of that medicine in this disease, precludes me from subscribing to this assertion. I have had many cases in which it has failed to cause even the absorption of the fluid, and very many in which after effecting its removal, it left the lung in a condition very different from that of health; in a majority of the cases recorded in this paper, mercury had been freely exhibited before the patients came under my care; in several it was given by my own direction; in three, ptyalism was produced and kept up, but nevertheless the fluid was not absorbed. To justify the assertion that all curable cases of empyema may be cured by mercury, without paracentesis, or, in other words, that no case of empyema which cannot be cured by mercury is curable by the operation in question, it would not be sufficient to show that paracentesis had not succeeded in curing any case where mercury had failed, unless it were also shown that at the time of its performance no such changes had taken place in any of the thoracic viscera, as rendered a cure impossible. Indeed, I am unable to conceive how such an assertion could be proved, for even if the whole of those thirty-five cases were perfectly cured, that is, not only that the fluid was removed, but that the lungs were restored to health and soundness—which is not alleged to have been the case—the point asserted would even then be rendered probable only, because other cases might still occur, in which mercury would fail; but it is not stated that these cases were so perfectly cured, and in the absence of evidence, or even any statement to that effect, we can only receive this assertion as the expression of the opinion of the late Dr. Hope, an opinion which we know to be contravened by the reports of many of the cases which have been published as cured. They state, that the shoulder of the affected side had dropped, that the chest was left contracted, and that the spine was deformed; facts which clearly indicate an accommodation of the bony parietes to a diminution in the size of the lungs, and lead us to the inevitable conclusion, that the lungs, so far from being cured, were too much injured to expand to their former dimensions." 207.

The author gives two striking examples of the extent of mischief sometimes done to the lungs of patients said to be cured of effusions into the chest by absorption. One was the case of a woman, aged 37, whose left side was much flattened, dull on percussion, and without even the feeblest sound of respiration. She had suffered some years before death from an attack of pleurisy, of which she had been cured. On examination of the body, the left lung was found considerably diminished in size, impervious to air, condensed into a dry homogeneous mass, and retained in contact with the ribs by a thickened and almost cartilaginous pleura: no tubercles were found in the lungs. In the other case, it does not appear that the patient was considered to have been *cured* of a pleuritic affection. The lower two-thirds, however, of the right lung were adherent to the ribs and condensed into a dry mass from the effects of this disease. The writer thinks that these cases are more common than is generally supposed, and that "they show that patients may be sent out of an hospital, apparently cured of empyema and hydrothorax, because the fluid has been absorbed, but who must suffer all their lives from the loss of one lung, and they prove most clearly that absorption of effused fluid is not necessarily a cure."

After arguing the propriety of having recourse to paracentesis, when mercury carried to salivation has failed to cause the removal of the fluid, in order to prevent this serious injury to the lungs, Dr. Roe alludes to the opinion of Broussais and Dr. C. J. B. Williams, "that retention of pus, possibly of serum, for a long time in the pleura, leads to the development of tubercles in the lung of the opposite, or even of the affected, side," as affording an additional argument in favour of an early operation. He combats the opinion entertained by some medical men that paracentesis, though necessary in empyema, is wholly unnecessary in hydrothorax, because those cases which cannot be cured by antiphlogistic means may be cured by generous diet and tonics, by the fact that Dr. Boyd, in the space of three years, at the St. Marylebone Infirmary, opened the bodies of 24 persons who died of hydrothorax, without tapping having been employed. The objections to paracentesis are reduced to this—"that it inflicts a wound; whilst in favour of it, it may be said, that in empyema it at once removes a noxious fluid, and does far less injury to the constitution than the absorption of purulent matter; that in both empyema and hydrothorax, it immediately relieves the lungs from the effects of pressure, and accomplishes that which internal medicines cannot, in the majority of cases, effect in weeks; and by its early employment, those irremediable changes already noticed are anticipated, and every chance is afforded for the complete restoration of the lung; that it removes that distention of the pleura, which paralyses the absorbents, and if inflammation be overcome by suitable remedies, it will effectually cure these diseases, provided the lungs are sound, and that, without inducing any evil consequences."

The author mentions the mode which Nature often adopts for evacuating the fluid, by the pus making its way between the intercostal spaces and bursting externally, and also through the lung itself into a bronchial tube and an interesting case is given in which a cure was spontaneously effected by the latter method. The cases in which it is considered that the operation should be performed, are serous effusions forming so rapidly as to endanger life; cases of empyema; serous effusions in persons of delicate health after the usual treatment has failed or been neglected; and mechanical hydrothorax, for the purpose of relieving the difficulty of breathing and prolonging life. The author details a case of the latter disease in which life was certainly prolonged seven months by the operation. We find the following excellent remarks on the conditions of success.

"Whether the operation shall materially assist in curing cases of simple pleuritic effusion, or afford merely temporary relief, depends on the time at which it is performed. To be successful, it is indispensably necessary that it should be employed before either the constitutional powers of the patient are too much reduced, or the thoracic viscera have undergone irremediable organic changes: for in the former case the absorbents cease to perform their functions, and therefore cannot prevent the re-accumulation of fluid after it has been removed; in the latter, a perfect cure is impossible. It is only when the lung is in a condition to expand to its full size, according as the pressure upon it is withdrawn, that the cure is effected without any visible alteration of shape in the diseased side. But when the operation is delayed till the lung has become atrophied, condensed, bound down by adhesions, or in any other way prevented from at once expanding sufficiently to meet the ribs, the shoulder becomes depressed and the side contracted, in order to bring the pleuræ into contact with each other: the body is then deformed, and the original capacity of the lung is very much diminished. When the lung is so much reduced in size that the pleura investing it cannot be brought into contact with the costal pleura, a cure is impossible: for a space must intervene between them, into which pus or serum will continue to be secreted, and the operation will be required again and again, till the patient dies from exhaustion. Under such circumstances, paracentesis cannot be looked upon as a curative measure, and therefore should only be employed to relieve distress of breathing." 220.

Dr. Roe attempts to fix the precise period after which the operation ought not to be delayed, which he makes about three weeks. We should have thought that the author's experience of this disease, and of its variable progress influenced by the state of the constitution and the action of remedies, would have shown him the futility of fixing upon any precise period for resorting to paracentesis. In several of the successful cases in the tables furnished by our author, the operation was delayed beyond three weeks. He states, however, that no case occurred in his own practice, in which, after a lapse of five or six weeks from the commencement of effusion, a patient was *perfectly cured.* The changes produced in the lung after this period, and sometimes before it, were irremediable. Dr. Roe gives the credit of the invention of the grooved exploring needle to Sir B. Brodie; but Dr. Watson ascribes it, and we believe justly, to the late Dr. T. Davies, of the London Hospital. The author advocates the plan of closing the wound after the operation, and repeating it if necessary, in preference to making a fistulous orifice by introducing a piece of elastic gum catheter, a point in which we decidedly concur with him.

Dr. Roe mentions a diagnostic mark of this disease, which he believes has not been hitherto described. It consists, in a marked degree, of fulness or even protrusion of the infra-clavicular region on the affected side; this often exists to a remarkable degree, and it is generally associated with increased resonance of the voice in the same situation. Are we indebted to Dr. F. Bird, or to the author, for this diagnostic mark ? the paper leaves us in doubt. He does not regard the bulging of the intercostal muscles as a constant or a frequent sign of the presence of fluid in the chest, and agrees with Dr. Stokes, who is of opinion that it only takes place in cases of purulent secretion. He believes that the protrusion does not depend on mechanical pressure, and is indicative of the quality, not of the quantity, of the effusion, but no attempt is made to explain its cause. Is not the bulging influenced a good deal by the thickness of the false membrane lining the costal pleura? when thin it must yield to pressure and when thick resist it. Dr. Roe very properly cautions the practitioner not to attach too much value to the side on which the patient lies as indicating the seat of effusion, and refers to a case in which death was produced by paracentesis having been performed on the healthy side from this cause. This ought not to happen, for no operation should be performed unless the other signs clearly indicated the seat and nature of the disease. The left side of the chest is more frequently affected than the right, in the proportion, as estimated from the cases in the paper, of seven to five. A table is given shewing the results of the operation in those cases in which it has been employed under the author's observation or that of his friends. Of these 24 cases, 18 recovered and 6 died. Nine of these were cases of empyema, of which 8 recovered and 1 died. Thirteen were cases of inflammatory hydrothorax, of which 9 recovered and 4 died. One was a case of mechanical hydrothorax and the patient was relieved. One was a case of pneumothorax and fatal. Dr. Roe also gives some notes of the result of this operation, collected by Mr. B. Phillips. Of 122 cases, 88 were cured: 31 were cases of pyothorax, of which 26 were cured, and 9 cases of hydrothorax, of which 6 were cured. The paper is followed by a long appendix containing the particulars of the 24 cases given in the table. Our analysis of this communication has been so full that we must refer those of our readers desirous of further information to the volume itself. It is remarked, in conclusion, that these cases "will show that the success of the operation was directly in proportion to the shortness of the time which intervened between the accumulation of the fluid and the performance of the operation, and that when it was unsuccessful the chief cause of its failure was its being postponed until too late a period."

Our author's experience of paracentesis thoracis, in cases of effusion into the chest, appears to have been unusually large, and the results which he has here detailed cannot fail to be useful in determining the doubtful questions of treatment. The propriety of operating in cases of empyema as soon as the presence of pus is detected, is pretty generally admitted; and Dr. Roe's observations show that, even in cases of hydrothorax, delay may be injurious, and lessen the chances of perfect recovery. We think, however, that the valuable information which this paper contains might have been presented to the Society in a less diffuse form. There is a good deal of repetition, and even common-place observations which add unnecessarily to the length of the paper.

XIX. ACCOUNT OF A CASE OF EMPYEMA WHICH RECOVERED AFTER REPEATED PUNCTURES OF THE PLEURAL SAC. By *Theophilus Thompson*, M.D.

The subject of this disease was a boy between five and six years of age, and Dr. Thompson was not consulted till the little patient had been ill two months, and had evident symptoms of considerable effusion in the right side of the chest. Mercurial ointment, quinine and decoction of chimaphila were tried for a few days, but as the effusion appeared to increase, an exploratory needle was introduced into the side, and pus detected. The operation of paracentesis was at once performed with a trocar, and fourteen ounces of greenish-yellow pus were withdrawn. The wound was afterwards closed. The boy was relieved for a time, but in three days it became necessary to repeat the operation, when a pint of matter was discharged. Ten days afterwards twenty more ounces were removed, and again on the 21st of July, at the end of eleven days, a fourth operation was performed, and twenty-two ounces of thick matter were rapidly withdrawn. He improved in strength, and on the 28th the wound re-opened and gave exit to about four ounces of pus. A discharge continued to take place from one or other of the orifices made in the operations for several months. In December, the discharge, which had previously averaged two ounces in twenty-four hours, was reduced to an ounce, and after the application of ung. hydr. nitr. ox. to the opening for a few days, ceased altogether, but returned on the discontinuance of the ointment.

"It was obvious, on the one hand, that any attempt to close the orifice would lead to injurious results, and on the other, that the fistulous opening might remain for life, unless some measure could be adopted to effect the gradual but complete emptying of the sac, and the approximation of its sides. We therefore determined cautiously to dilate the opening, and with this view Mr. Robarts prepared a plug, consisting of a piece of sponge, which had previously been firmly tied round with packthread, and saturated with wax.

"This plug was introduced at night, on the 31st of December, and when removed on the 2nd of January, was followed by a copious discharge of pus. In a few days, the aperture having again contracted, another plug was introduced, and the next day withdrawn, when about six ounces of pus were discharged in a jet.

"As the results of this plan proved so encouraging, the plug was again introduced on the 22nd of January, and removed the next day, when half an ounce of matter, still inoffensive, was removed." 278.

After this period there was no fresh discharge, the orifice permanently healed, and the boy has remained perfectly well.

The author, in his observations on this case, takes the same view of the inutility of remedies and the necessity for a prompt operation in cases of empyema, as that advocated by the writer of the preceding paper,—a view which is sanctioned by our best practical physicians. He adopted also the same practice as that pursued by Dr. Roe, of repeating the operation in preference to leaving a canula in the wound.

We are at a loss, indeed, to find sufficient novelty in the case to account for a place being allotted to the paper in these Transactions. The chief point of interest, was the successful attempt to promote the gradual contraction of the suppurating cavity by dilating the fistulous opening, so as to permit a free discharge of the pus, the credit of which practice the author justly gives to Mr. Robarts, who originally requested Dr. Thompson's assistance in the case.

### XX. Observations on the Omental Sacs which are sometimes found in Strangulated Herniæ, completely enveloping the Intestine, with Cases and Dissections; to which has been added, a Table of all the Strangulated Herniæ operated on at St. George's Hospital, in 1843-44. By *Prescott Hewett*, Esq.

The author remarks that, in an operation for strangulated hernia, the intestine is not unfrequently found surrounded by omentum, which appears to form a second sac; but, with a little care, it may be unfolded, and the intestine thus easily laid bare. But cases in which the intestine is contained in a *complete sac,* formed by the omentum, which it is *absolutely necessary to divide*, to reach the gut, have been rarely met with. After alluding to the imperfect notices of this subject to be met with in authors, he mentions that complete omental sacs were found in four cases out of thirty-four operations for strangulated hernia, performed at St. George's Hospital, in 1842-43; of these four cases, two were femoral, one inguinal and one umbilical.

"The formation of these sacs is attributed, by Richter, to the firm agglutination of the margins of the omentum which has surrounded the bowel. To this explanation of Richter's, which does not appear to be applicable to the majority of cases, the two following explanations of the manner in which these sacs are, in some cases, formed, have been added.

"1st. The gut, *completely enveloped* by the omentum, passes through the ring, and the omentum thus disposed round the intestine, becomes attached to the circumference of the neck of the hernial sac; this omental pouch is subsequently distended by the intestine, and thus forms a complete lining to the hernial sac.

"2nd. An epiplocele takes place, and the portion of omentum which is protruded becomes altered in structure, and its folds firmly united to each other by the effusion of lymph; but, within the abdominal cavity, in the neighbourhood of the ring, the folds into which the omentum has been drawn may not be agglutinated; they will thus leave spaces into which a knuckle of intestine may insinuate itself, pass through the ring, and form for itself a bed in the altered mass of omentum which is in the hernial sac. It may happen, that two or three portions of gut may slip into the different spaces left between the folds of the omentum, and subsequently form for themselves separate pouches. Several separate sacs, with narrow necks, may be thus found in the omental mass which is in the hernial sac." 285.

Once formed, these sacks may attain an immense size. In one case, the sac measured six inches in length, and eleven inches in circumference, at its broadest part.

"The omentum in which a sac has been formed may, in the course of time, especially if it is irreducible, become altered in structure, either by the effusion of lymph, or by a deposition of fat which takes place in the walls of the sac.

"By this alteration of structure the thickened sac may, in an operation, become the source of very great difficulties. In Case 4, it will be seen that so great was the accumulation of fat in the layers of the omentum forming the sac, that its walls were, in some places, more than an inch thick. In this case the deposition of fat had principally taken place towards the lower part of the sac; at its neck the omentum was but slightly altered in structure. The incision made at the time of the operation corresponded to this part of the omentum, so that, when the hernial sac was laid open, the gut appeared to be covered merely by a band of omentum, which was easily divided, and the gut reduced; after which the sac was ascertained to be, with the exception of this slightly thickened band of omentum, quite empty; but no satisfactory explanation could be given of the nature of the thickened mass which still remained in the scrotum. A careful dissection, made at the *post-mortem* examination, proved that this mass consisted of the thickened omental sac, above referred to." 286.

These omental sacs may either lie loose in the cavity of the hernial sac, or the two sacs may have contracted more or less extensive and firm adhesions with each other. In one case, the omental sac was quite free from adhesions; in another, the two sacs adhered firmly to each other at the neck, in the whole of their circumference; and in Case 4, the two sacs were firmly united to each other, throughout their whole extent, by a firm, close, cellular tissue.

The author remarks that these cases "naturally give rise to a question of great practical importance, as to what course ought to be pursued where the hernial sac appears to contain thickened omentum only. In such cases the omentum ought to be drawn out and carefully examined, to see that it does not form a sac containing a portion of intestine. If the omentum is thickened, and firmly united to the neck of the hernial sac, throughout its whole circumference, a longitudinal incision should be carefully made in the whole length of the thickened omentum, to ascertain that it does not form one of these omental sacs. The enormous thickness which the walls of an omental sac may present, must here be borne in mind. The precaution of introducing the finger to ascertain that the neck of the sac is free, is, in these cases, particularly necessary."

The intestine contained in an omental sac may be united to its internal surface. In one case the intestine was firmly united to the neck; in the three other cases, the intestine was free from adhesions. The neck of the omental sac may become the sole cause of strangulation; of this Mr. Hewett mentions a well-marked example, in which Mr. Hawkins was obliged, after having freely divided the neck of the hernial sac and the ring, to divide the neck of the omental sac before the gut could be reduced. Had the practice of reducing the hernia without opening the hernial sac been followed in this case, the gut, still strangulated by the omental sac, might have been reduced, and a fatal termination been the consequence. Such cases as these, the author considers, are a strong argument against the practice of reducing a hernia without opening the hernial sac. Upon this point we beg to differ from him. The argument applies with equal force as an objection to the employment of the taxis, but the complication is, as Mr. Hewett admits, very rare, and we think that no surgeon should be deterred by the liability to it, from resorting to the taxis, or dividing the stricture external to the sac. We believe, indeed, that the reduction "en masse" is more likely to occur from the taxis before the operation than when the integuments of the sac are divided, and the parts are more fully under the observation of the surgeon. The division of the omental sac, it is remarked, may be followed by hæmorrhage, the vessels being sometimes enlarged. There can be no difficulty in securing them if necessary. In conclusion, the author points out the importance of *carefully* examining every portion of omentum which is in a hernial sac, so as to ascertain that no knuckle of intestine is contained within its folds, before it is returned into the abdomen, left in the sac, or removed altogether. The four cases are well described, but we have not space for the particulars.

The table of strangulated herniæ includes 34 cases. Of these, 25 were cured, and 9 died. In all, the sac was opened; in some, an attempt was made to reduce the hernia without opening the sac, but unsuccessfully.

We believe that most surgeons who have had experience in operations for hernia are acquainted with the complication described in this paper, but as the subject has been imperfectly noticed by writers, Mr. Hewett's observations will be read with much interest.

### XXI. Account of a Case of a Dissecting Aneurism of the Aorta, Innominata, and Right Carotid Arteries, giving rise to Suppression of Urine and White Softening of the Brain. By *Robert Bentley Todd*, M.D. F.R.S.

The subject of this disease was Mr. T.———, æt. 37, a stout muscular plethoric man, who was suddenly seized on the 16th of February, whilst sitting at dinner, with a fainting fit. He quickly recovered, but complained of violent abdominal pain and nausea, with pain in the back. Vomiting ensued. Next day he complained of violent pain in the loins, extending to thc groins and testicles, which was relieved by cupping. Great drowsiness; urine scanty and high-coloured. On the 19th, *no urine had passed for forty-eight hours*. Dr. Todd found him lying on his back, with all the aspect of a man labouring under an oppressed brain. When aroused he answered questions rationally, but immediately relapsed into his previous drowsy state. There was evident paralysis of the left side of the face, and also imperfect paralysis of the left upper and lower extremities. The pulse was full, but compressible; but there was a marked difference on the two sides. *On the right it was small and feeble.* On examining the heart, the first sound was accompanied by a bellows-sound, distinctly audible in the direction of the arteria innominata. It had a peculiar rumbling character, as if the blood had to encounter a considerable obstacle. On enquiry, he stated that he had always enjoyed good health, but that he could not breathe as freely on the left side as on the right. The treatment consisted chiefly of cupping and blisters to the back of the neck, purging and afterwards diuretics, the hot-air bath, and subsequently stimulants. On the 26th, the action of the kidneys was restored, and the paralysis slightly diminished; but the patient evinced an alarming tendency to sink. On the next day, whilst raised in bed, having just finished drinking, he was seized with a slight convulsion, and fell back dead. The body was examined on the 29th. Surface exsanguineous. Very little blood in the divided vessels of the scalp. The right side of the brain decidedly paler than the left. When the hemisphere was cut into, there was a total absence of bloody spots, which however existed in their ordinary number in the left hemisphere, which was perfectly natural.

"On examining the cut surface of the centrum ovale on the right side, it presented the appearance as if it had been worm-eaten in patches. Each patch was from half an inch to an inch in diameter. It had the same colour as the surrounding brain substance, but was evidently diminished in consistence: the slightest lateral friction with the edge of the knife completely disarranged it; and when a stream of water was poured upon it, it was broken up into shreds which floated in the water. These patches were very numerous; they were found in all that part of the hemisphere which is above the fissure of Sylvius; each was surrounded by cerebral substance of nearly natural consistence. Many of the patches were superficial, and involved portions of the grey matter of the convolutions, which exhibited the same degree of softening. They were all perfectly free from admixture with any foreign material, such as pus, or with blood. I searched for the former by the aid of the microscope, but could find no trace of it. Nerve-tubes were seen in their ordinary number, which seemed easily broken up and very varicose.

"The right half of the fornix, and the septum lucidum, were likewise extremely soft.

"The paleness of the grey matter of the convolutions on the right side particularly attracted notice. No appearance of red blood was visible to the naked eye. On the left side, the colour was perhaps a little paler than is natural.

"The bloodless state of the right side was strikingly evinced by the extreme paleness of the choroid plexus on that side, while that of the left side retained nearly its natural colour." 315.

The author remarks :—

"These appearances at once explained the paralysis of the left side, under which our patient laboured. It was plain, from the peculiar exsanguineousness of the whole hemisphere, that it must have been deprived of a large proportion of its usual supply of blood; and that that portion of it suffered most which derived its nutriment from the middle cerebral artery in the fissure of Sylvius. In fact, this side of the brain was thrown upon the branches of the vertebral artery and the anastomosing branches from the opposite side for its supply of blood. The improvement which took place in the paralytic symptoms during the last two days of our patient's life, denoted that the collateral circulation had begun to convey a larger amount of blood to the right side of the brain; and no doubt, had he lived longer, it would have been quite sufficient to restore the nutrition of that organ." 316.

No explanation of the exsanguineous state of one-half of the brain could be derived from an examination of its arteries : they were pale and empty, but free from disease. The chest was opened, and the pericardium found distended with blood; a layer of dark and recent coagulum, nearly an inch in thickness, surrounding the heart, which was larger and firmer than natural.

"The source of the hæmorrhage was found to be the aorta, which had given way, and allowed the blood to escape through a small fissure of its external coat, and of the portion of the pericardium which is reflected on it. This was situate about an inch beyond the ostium aortæ, and was large enough to admit a small goose-quill.

"The arch of the aorta at this part was evidently much dilated. All the coats, however, did not participate in the distension, for it was soon evident, on examination, that the outer or cellular coat was separated from the middle coat, for some extent, leaving a considerable space in which the blood accumulated.

"The aorta was laid open along its concave border. The semi-lunar valves were healthy. Several atheromatous patches were found in the valve of Lieutaud, in the arch, and in greater number in the descending aorta. In one of these patches, about half an inch beyond the free margin of the semi-lunar valves, ulceration had taken place. This ulcer was the starting point of a transverse rent, which involved the inner and middle coats of the artery, and through which the blood found its way into the sac, between the outer and middle coats.

"The separation of the coats, however, was not limited to the first portion of the aorta. It proceeded along the posterior surface and convex edge of the arch, involving the innominata artery, and, to a partial extent, the left carotid and subclavian. The middle coat of the aorta was split into two layers, between which the blood formed for itself a new channel, down to the abdominal aorta, in which it was evident, from the presence of recent coagula in it, that blood had recently flowed.

"A new channel was likewise formed by the splitting of the middle coat of the innominata on its outer and posterior part. This channel extended up to the lower part of the common carotid artery, and the blood accumulated there to such an extent as to obliterate the canal of the artery completely, and effectually to stop the ascent of the blood into it. Two channels were thus found in the innominata; one leading to the carotid, which was formed by the splitting of the middle coat; the other, the natural one, much diminished in size, through which the subclavian was supplied." 318.

All the tunics of the arteries involved in the laceration wore a healthy aspect, and without doubt the laceration was quite recent. By an oversight the abdominal aorta was not examined, but Dr. Todd thinks it highly probable, from the suppression of urine taking place so early after the seizure, that the new channel must have extended so low as to impede, for a time, the current of blood in the renal arteries. The kidneys were large and in the second stage of granular disease. Dr. Todd makes the following remarks upon the connection between the morbid appearances and symptoms experienced during life.

"It cannot be doubted that the laceration of the aorta and the formation of the new channel throughout its course was the cause of the fainting fit which ushered in his illness. The sudden diversion from the brain, of so large a portion of blood as that which one carotid artery is accustomed to supply to it, whilst the patient was in an erect posture, was alone a sufficient cause for syncope. And to this may be added the disturbance which the heart must have experienced from the sudden opening of the new channel. The severe pain in the back is explained by the forcible tearing up of the aortic walls, by means of the column of blood forced along it. Those who attend to the sensations which are produced in their own persons under the influence of particular changes, must have remarked the painful feeling which is perceived along the course of the abdominal aorta, when the heart's action is forcibly excited by a sudden emotion of the mind. How much greater must be the pain which is excited by the *laceration*, than that which results simply from the distension of the artery!

"The encroachment upon the calibre of the innominata by the formation of the new channel in it, accounted for the peculiarity of the pulse in the right wrist, which attracted our attention from the first. And the plugging of the carotid low in the neck, explained the laboured pulsation which was felt just below the obstacle.

"Not the least interesting point in the case is the obvious connection which existed between the softening of the brain and the stoppage of the circulation in the common carotid artery. We have seen that the patches of white softening were limited to that part of the brain which is supplied by the middle cerebral artery. This artery is the principal branch of the carotid within the cranium, and has a less free communication with the corresponding ramifications of the opposite side than any of the other arteries of the brain. Hence the parts supplied by it are more apt to suffer than those which are nourished by the other branches of the carotid.

"How strikingly illustrative, too, is this case, of the nature of that morbid change which is known by the name of *white softening*, or more properly *softening without discolouration*. There was not a particle of evidence derivable from the anatomical condition of the parts to prove the existence of inflammation. The softening depended upon the cessation of the circulation in the carotid as evidently as do cases of senile gangrene upon obstructed arteries in the affected limb.

"The internal carotid arteries of opposite sides do not anastomose with each other by any means so freely as do the external. This was illustrated by the fact that the circulation was very quickly restored in the branches of the external carotid, for the pulsations of the right temporal artery were all along distinct, and there was no appearance of any want of nutrition in the right side of the face.

"When the circulation is obstructed in the *internal* carotid artery, the chief source of supply to the brain on the same side is from the vertebral artery. That this collateral circulation is amply sufficient to prevent any injurious influence upon the functions of the brain, is abundantly proved by many recorded cases of spontaneous or artificial obstruction of the common carotid artery.* How is it that it was insufficient in this case? This question may be satisfactorily answered by referring to the narrowed dimensions of the innominata and right subclavian arteries, and the consequent imperfect supply of the right vertebral. In the cases above alluded to, in which no cerebral symptoms followed obstruction of the carotid, the course of the vertebral was free and unimpeded." 323.

As additional evidence of the gradual way in which the seeds of serious organic disease are sown without attracting attention, it is stated that he was twice passed as a healthy assurable life within a recent period.

We regret that we have been obliged considerably to abridge the particulars of this very interesting case.

### XXII. CASE OF ANEURISM OF THE EXTERNAL ILIAC, IN WHICH A LIGATURE WAS APPLIED TO THE COMMON ILIAC ARTERY. By *Richard Hey*, Esq.

The author first gives a brief account of the nine cases in which a ligature has been applied to the common iliac artery. Of these, six were unsuccessful, and three successful. The present case makes a fourth in which the patient recovered. When Mr. Hey visited his patient, the tumor had rapidly increased in the course of ten or twelve days from a very small size, accompanied with great pain in the course of the anterior

* "See the chapter on Carotid Aneurism in Mr. Hodgson's book on the Diseases of Arteries."

crural nerve. As the skin soon threatened to give way, the operation of tying the common iliac was determined on.

"The tumor now occupied the whole of the left iliac fossa, extending from below Poupart's ligament to within little more than an inch from the umbilicus; the vertical diameter was six inches, the transverse six inches and a half; the swelling also projected at least three inches from the plane of the abdomen. It presented all the usual characters of aneurism. The patient was placed on a mattrass, inclining somewhat to his left side, and his shoulders moderately raised.

"I commenced the operation by an incision which reached from about two inches and three quarters above the umbilicus to the base of the tumor, being about six inches in length, and moderately curved; this was afterwards extended by an angular continuation an inch and a-half in length; it was also exactly three inches to the left of the median line. The fibres of the external and internal oblique and transversalis muscles were successively divided; and the transversalis fascia having been readily raised with a director, was carefully opened, to an extent equal with that of the external incision. The peritoneum now protruded, but being greatly depressed and drawn towards the opposite side, I was enabled slowly to insinuate my fingers behind it, so as to separate it from its cellular attachment to the adjacent parts. The common iliac artery was easily reached, and its compression with the finger instantly stopped the pulsation in the tumor. A little time was occupied in scratching through the sheath of the artery; a common silver aneurism needle was now passed under the artery, armed with a double ligature of staymakers' silk, waxed. By holding aside the peritoneum and viscera, a momentary view of the artery was now obtained, and its complete isolation ascertained. The ligature was then tightened with the fingers close down upon the artery, when the pulsation entirely and finally ceased. The situation of the ligature was I believe an inch below the bifurcation of the aorta, or very little more." 329.

The time occupied in the operation was 25 minutes. The patient was much exhausted, notwithstanding the little blood lost. The wound healed favourably and the ligature came away on the 28th day. The author notices a source of anxiety in the after-treatment, viz. distension and violent spasm of the bowels accompanied with obstinate costiveness. This was found to proceed from the bowel being distended with an enormous mass of dry and hard fæces, the pressure of the aneurismal sac on the colon having prevented the contents of that viscus from descending into the rectum. The mass was with difficulty broken down and the patient relieved.

We sincerely congratulate the author on the result of his surgical skill in this case. It must undoubtedly tend to maintain the fame which has so long attached to the name of Hey.

### XXIII. Two Cases of Tubular Expectoration from the Bronchi, in the Adult. By *James Reid,* M.D.

The author, after noticing the rarity of this disease and that few cases of it are placed on record, describes two cases. In one, a married woman, æt. 28, after an attack of bronchitis followed by a chronic cough, expectorated several arborescent membranous substances, resembling casts of the minute bronchial tubes, for three days, the quantity gradually diminishing. She suffered from a recurrence of the attacks five or six times, at intervals varying from one to three weeks in extent, but each attack being preceded by a sense of suffocation. The patient lost flesh and strength, but recovered her health on removing to Devonshire in the summer. On her return to town in the winter she had a return of the complaint, which was again relieved by the expectoration of the arborescent tubular substance. There was no further relapse, and the patient died the succeeding winter of a disease unconnected with the chest. The second case was that of a barrister, æt. 44, who, after several attacks of cough, expectorated several portions of tubular substance, resembling plastic casts of the extreme bronchial tubes, accompanied with hæmorrhage. The expectoration of this solid matter and blood recurred at intervals, but subsequently ceased, and at the end of nearly two years there had been no return of the disease. It is a curious coincidence, that the brother of this patient was afterwards affected with the same complaint. The case is related by Dr. Watson in his published Lectures. The bronchial concretions seemed, with few exceptions, to be hollow, and to contain both air and blood. In the first case there was great dyspnœa, but in the second the cough was never accompanied by a feeling of suffocation. The author, we think, rightly attributes the bleeding to the partial detachment of the plastic substance from the turgid mucous membrane. He considers the disease to be chronic inflammation of the mucous membrane of the bronchial tubes of a specific kind, and that the exudation does not depend on the intensity of the inflammation, but on some peculiar action of the parts producing it. It may attack both lungs, or be confined to one, as in the author's second case. He thinks the prognosis favourable provided the malady is not complicated with phthisis or other serious disease. The higher the plastic formation takes place in the air-tubes, the more severe will be the symptoms. Dr. Reid does not recommend active treatment, but is disposed to rely more on the employment of ipecacuanha, squills, and other mild expectorants, conjoined with light diet.

### XXIV. A Tabular View of the Seat of Tubercle in one hundred and eighty Cases of Tubercle of the Lungs in Children, with Remarks on Pulmonary Phthisis in the Young Subject. By *P. Hennis Green*, M.B.

In this paper the author proposes to indicate a few of the peculiarities which distinguish infantile consumption from the phthisis of adults. He gives a table of 180 cases of thoracic tubercle for the purpose of furnishing data for the history of pulmonary consumption in children. He remarks, " the main character which distinguishes the phthisis of children from that of adults is this,—in children, the tubercular deposit occupies a much larger surface of the lung, is more rapidly secreted, and is complicated with tubercular disease of other organs more frequently than in the adult. Hence children often sink under phthisis before the complaint has arrived at its third stage; while, on the other hand, the modifications produced by an extensive diffusion of tubercular matter often render the diagnosis of the disease obscure and difficult. In addition to this character we have the peculiarities occasionally induced by excessive tuberculization of the bronchial glands, giving rise to bronchial phthisis, a form of disease altogether confined to the child."

The author notices an important modification which should guide the practitioner when he seeks to determine the existence of cavern in young children, viz. that under five years of age, the cavernous excavation is generally seated in the lower or middle lobes, and is almost always confined to one side of the chest. To show that the general diffusion of tubercular matter forms a striking characteristic of phthisis in children, Dr. Green compares some of M. Louis' results with those deducible from his table.

"In 358 cases of phthisis in adults, M. Louis mentions the existence of tubercular matter in the brain or its membranes only once. In the bronchial glands, tubercles were found in about one-fifth of the cases; in the mesenteric glands, in one-fifth; in the liver, only twice; in the kidneys, five times in 170 cases: on the other hand, ulceration of the larynx existed in one-fourth,—ulceration of the bowels in five-sixths of the cases.

"The history of phthisis in children presents us with very different results. The brain was affected in one-ninth of the cases; the bronchial glands, in 100 out of 112; the mesenteric glands, in one-half; the liver, in one-ninth; the kidneys, in one-eighteenth; but ulceration of the larynx occurred only once; ulceration of the bowels, sixteen times in the 112 cases." 356.

The physical signs are rarely as well marked as in the adult, and the young child frequently dies before the practitioner is able to decide whether the lung be actually the seat of cavern or not. The cause of this is that, in children, "the tubercular matter is widely diffused, and has implicated many important viscera; in the brain, it may excite hydrocephalus or meningitis; under the serous membrane of the chest, pleurisy; in the abdomen, peritonitis; in the intestines, tubercular ulceration. These complications rapidly undermine the resisting power of the little patient; diarrhœa sets in, and death ensues long before the period at which a fatal termination takes place in the adult."

Infants and children under five years of age hardly ever expectorate. They swallow every thing that comes up into the mouth from the lungs. Hæmoptysis is an exceedingly rare symptom. The symptoms which constitute hectic fever in adults are seldom present together in any marked degree.

The bronchial glands were more or less affected in one hundred out of one hundred and twelve cases. In a few of these cases only were the glands sufficiently enlarged to produce symptoms through their mechanical effects, or by communication with caverns in the lungs and the bronchi, and in such cases the term bronchial phthisis should be confined. Understood thus, this form of phthisis is peculiar to children, and attended with very characteristic symptoms; but it is not, as some writers assert, of frequent occurrence.

"The enlarged bronchial glands may act mechanically on the neighbouring organs contained in the chest, or they may perforate them. Hence a variety of symptoms, depending on the position or function of the injured part.

"The aorta and pulmonary artery, the vena cava, or the pulmonary veins, may be compressed by the tuberculated glands, and the flow of blood more or less impeded. M. Tonellé has related a case in which the superior cava was completely obstructed, and I have seen one where the pulmonary artery was perfectly flattened between two enormous glands. From the compression of vessels may arise pulmonary apoplexy, fatal hæmorrhage, effusions of serum, or symptoms closely resembling those of organic disease of the heart. The trachea, bronchial tubes, and lungs, may be compressed, and in such cases the symptoms will vary considerably, according to the seat and extent of the mechanical lesion. When the ganglions act on the lower portion of the trachea, MM. Rilliet and Barthez have noticed the existence of a loud, sonorous ronchus, which persists for a considerable length of time. In other cases, the pressure on the large bronchial tubes causes more or less feebleness of the respiratory murmur, which is remarkable in being intermittent.

"Pressure on the eighth pair of nerves or its branches is often attended by very peculiar modifications of the voice and cough. The former is hoarse or occasionally subdued, and even lost; or the hoarseness and loss of voice may alternate. The cough, also, is frequently hoarse, or occurs in fits which bear a close resemblance to those of hooping-cough, but are not followed by vomiting; or the fits may simulate accesses of asthma, with great oppression of breathing, anxiety, agitation, congestion of the head, and cold, viscid sweats.

"The enlarged or softened glands give rise to another order of symptoms, by perforation of the neighbouring parts. Thus fatal hæmorrhage may arise from perforation of the pulmonary artery; pneumo-thorax from perforation of the lung; difficulty of deglutition and accesses of cough on swallowing from perforation of the œsophagus; but we should observe that these symptoms may equally depend on the presence of tubercular matter or of a cavern in the lungs." 365.

On the subject of diagnosis of this form of phthisis, the author remarks:

"Whenever a child presents several of the rational symptoms of consumption, without our being able to detect any physical signs of the presence of tubercles in the lungs or abdomen, we have good reason to suspect that the bronchial glands are tuberculated. As long as the case continues to present this simple aspect we cannot go beyond suspicion: but it rarely happens that the glands acquire a considerable degree of development, without acting on the surrounding parts or tissues. As these become successively involved, we have a series of varying symptoms which could not arise from any other source. The eyelids become œdematous, and in proportion to the degree of pressure on the vena cava, the œdema extends to the whole of the face, which is sometimes pale, sometimes tinged with venous injection. This œdema will appear and disappear several times during the course of the disease. The cough suddenly changes its character, and occurs in fits, like those of hooping-cough; the voice gets hoarse, and for days may be altogether lost; fits of asthma or of suffocation, as if the heart were diseased, occur. On examining the chest, we heard a loud sonorous ronchus, which persists for a length of time, and then disappears, or is replaced by other *râles* of an anomalous character. When these symptoms are superadded to the rational signs of phthisis, we can have little hesitation in deciding that they arise from tubercular enlargement of the bronchial glands." 366.

We find nothing new on the subject of treatment. Dr. Green objects to emetics, lest irritation of the abdominal viscera hasten the deposit of tubercle in the abdomen, to which the patients are already too prone.

We much regret that we have been compelled to give so condensed an analysis of this excellent paper. Much, very much as Louis has done, it is evident that he has not exhausted the subject of phthisis, and the accurate information presented to us by Dr. Green will be received as a valuable addition to our history of the disease at the period of infancy.

XXV. Case of Tumor in the Right Hypochondrium, occurring after Injury, from which a large quantity of Fluid resembling Bile was repeatedly withdrawn by the Operation of Tapping. By *William Robert Barlow,* Esq.

On the 28th of August, a strong healthy man, aged 54, a thatcher, injured himself by lifting a heavy ladder. He complained of so much pain in the region of the liver, that the author apprehended a rupture of that organ. He was faint, in a cold perspiration, and the pulse was scarcely to be felt. He recovered sufficiently to be removed home. By depletion, calomel and opium, &c. the pain was relieved, but his motions presented no appearance of bile. Sept. 15, a swelling, the size of a walnut, was observed over the region of the liver. This gradually increased; he suffered great pain from distension; and from the swelling being limited to the right hypochondrium, and no bile having yet passed by the bowels, Mr. Barlow felt confirmed in the opinion, that a rupture of the liver, involving the biliary ducts, had occurred. Oct. 9th. He tapped the swelling and drew off seven quarts of fluid, which, from the colour and taste, appeared to be pure bile. The patient was relieved, but the fluid recollected, and on the 21st, six quarts more of fluid were removed. This on analysis was found to be a serous fluid mixed with bile. He was again tapped, on the whole six times, the last occasion being on the 26th of November. On the following day, after a dose of castor-oil, some bile was observed in the motions. The swelling subsided, the stools became natural, and by the commencement of the February following he had completely regained his health. The editor has appended to this very interesting case the particulars of another bearing a close resemblance to it, related by Dr. William Thomson of Edinburgh.

XXVI. Peculiar Case of Gelatiniform Cancer, in which nearly all the Organs of the Body contained Colloid Tumors; with the Appearances on Dissection. By *John C. Warren,* M,D.

The author states that colloid cancer is described by authors as occurring in one single mass, usually of considerable size, but in the case here described, the disease was diffused through nearly all the textures of the body, without presenting any one considerable mass. The case is also remarkable in exhibiting a union of the three admitted forms of malignant disease, scirrhoma, cephaloma, and gelatinoma. The patient was a young man, aged 25, but as the chief interest of this case is connected with the pathological appearances, it will be sufficient to mention, that on examination of the body—its surface was studded with subcutaneous tumors about the size of a hazel-nut. They were composed of small granulations, constituted by sacs containing a substance which appeared at first view to to be wholly gelatinous, but which, on being divided, discharged a small quantity of viscid fluid. The colour was a mixed grey and red; they were slightly transparent. Similar tumors were found in the thyroid gland, diploë of the skull, ribs, muscles, mediastinum, heart, lungs, mesenteric glands, stomach, kidneys, testes, &c. There were also tumors of scirrhoma in the liver and of cephaloma in some of the lymphatic vessels. Dr. Warren mentions that the tumors soon lost their transparency on immersion in alcohol. This is not the character of colloid cancer, and we are inclined to suspect that the disease consisted of cysts containing an albuminous fluid, which are occasionally developed in connexion with carcinoma. The case however is interesting, and thanks are due to the Transatlantic Professor for the communication.

### XXVII. CASE OF NECROSIS OF THE LOWER JAW RECOVERED FROM WITHOUT DEFORMITY. By *William Sharp*, F.R.S.

About two-thirds of the entire lower jaw were removed by a slight operation. The case offers, however, no features of particular interest, and is much less remarkable than a case related by Mr. Perry, in the twenty-first volume of the Transactions, in which the whole of the inferior maxilla was taken away.

### XXVIII. REMARKS ON THE PATHOLOGY OF MOLLITIES OSSIUM, WITH CASES. By *Samuel Solly*, F.R.S.

The attention of the author was called to this rare disease, by two cases, in which he had the opportunity of tracing its progress during life, and examining the morbid appearances after death. After adverting to the opinion of Dr. Cummin, who, in treating of mollities ossium, calls it the rickets of adults, and to that of Mr. Curling, who, in a minute account of this disease,* alluded to by Mr. Solly, has regarded it as an atrophy of bone; he describes the case of a young woman, aged 29, who having enjoyed good health in early life, began to decline at 19, after an attack of scarlet fever. She suffered pains in the back, passed urine with a whitish sediment, and her spine began to yield. She had pain in the head, became maniacal, and was admitted into St. Luke's Hospital. Whilst there she lost the power of standing, and screamed violently, as if in pain. Her head was observed to be enlarged, and her eyes to project, caused no doubt by the thickening of the walls of the orbit. She was discharged incurable, and after some time taken to Hanwell.

"At the time she was received into this asylum she was much emaciated, and enfeebled, with loss of power in her lower extremities; and two or three months before her death, the bones of the extremities were observed to lose their natural direction, and become curved; subsequently, fractures took place from the slightest causes. She suffered excruciating pain during the whole time she was in the asylum, which she referred to her bones; she did not suffer from spasm of the muscles, as many of these cases do, and the urine, during the whole time she was at Hanwell, was clear and natural. Her appetite was good, and all the functions duly performed, with the exception of the catamenia. Large doses of morphia and other sedatives were administered, to procure sleep and relieve pain. Her mental aberration was extremely slight. Her sufferings were terminated by death on the 28th October 1842." 441.

---

* Med. Chir. Transactions, Vol. xx.

On examination of the body, the author found, in addition to deformity of the spine and chest, fractures of the left humerus, both clavicles, radius, and both femurs (the left in two places). All the bones of the extremities could be fractured with the slightest force—by merely pressing them between the finger and thumb, they gave way and cracked like a thin-shelled walnut.

"A longitudinal and transverse section of the long bones showed that the osseous structure of the bone was nearly absorbed, a mere shell being left. The interior was filled with a dark grumous matter, varying in colour from that of dark blood to a reddish light liver colour. I could not detect any pus globules in it under the microscope. The bones of the vertebral column and ribs were similarly affected; cranium very much thickened, and at least half an inch in diameter, so very soft as to be easily cut with a knife, and very vascular; the two tables were confounded, and the diploë obliterated. Thin slices of the cranium, under the microscope, showed that a considerable alteration had taken place in its ultimate structure. The laminated structure of the outer and inner tables was extensively absorbed. The Haversian canals enormously dilated, and the osseous corpuscles diminished in quantity." 442.

The second case was also that of a female, thirty-nine years of age and married. She suffered severely from the pains usually considered rheumatic; her frame became greatly distorted, much in the same way as represented in the well-known case of Madame Sapiot, many bones were fractured, and she ultimately died in St. Thomas's Hospital from asphyxia. During life, her urine was found on examination to contain a large quantity of phosphate of lime—between three and four times the quantity of healthy urine. The body was examined, and in addition to the thinning of the bones which the nature of the case would lead us to expect, the right lung was so compressed as to be almost impervious to air. The left kidney contained a large calculus, consisting solely of phosphate of lime. Sections were made of several of the diseased bones, and the alterations thus exhibited, consisted chiefly of absorption of the osseous tissue, its place being supplied with a red substance contained in cells more or less vascular. The red matter was examined under the microscope by Mr. Birkett and Mr. Simon of King's College. The latter gentleman states:

"My examination was not at all satisfactory as to the ultimate nature of the disease. There was great excess of the natural fatty matter, and disproportion of the medullary cells to the substance of the bone; in parts there was apparently extravasation of blood, which may have arisen from violence. I was unable to discover any new cell formation, at least any mature one; cytoblasts were exceedingly plentiful, so as to suggest the probability that some such formation was in progress, but nothing further, with the exception of some two or three apparently detached young fat cells. Decidedly there was no show of growing cartilage." 455.

Mr. Solly, after alluding to some of the opinions as to the nature of this disease entertained by writers, remarks, "after a careful consideration of all the facts, but especially by comparing the appearances after death, with the symptoms during life of this awful disease, I am led to believe that it is of an inflammatory character. That it commences with a morbid action of the blood-vessels, which gives rise to that severe pain in the limbs, invariably attendant on this disease, but more especially in its commencement, and exhibits itself after death by an arterial redness of the part. The absorbent vessels are at the same time unnaturally excited, and the earthy matter of the bone is absorbed and thrown out by the kidneys in the urine, which excretion is sometimes so abundant, as we have seen in the last case, that it clogs up the calices and pelvis of the kidney, and forms there a solid calculus."

The cases related by Mr. Solly are interesting and well deserving of record, but it will be perceived that he has not succeeded in throwing much light on the nature of mollities ossium. We believe him to be quite wrong in describing it as of an inflammatory character. The altered state of the osseous tissue is essentially different from that which commonly occurs in bone, as the consequence of inflammation. The appearances of extravasation of blood and of injected vessels, most probably arose from the disturbed state of the bones—the fractures and distortions; and the excessive deposition of fatty matter would necessarily be accompanied with increased redness of the part. The whitish sediment generally remarked in the urine of persons affected with this disease, has long been suspected to consist of the phosphate of lime; and the fact is established by the chemical examination of the urine in the second case in this paper. Mr. Solly states " the microscopic examination of this matter, showing cell development in its various stages, confirms my impression that it is an adventitious morbid product, and not simply the fatty matter of the bone altered by the effusion of blood into it."

It is clear from the account given by Mr. Simon, above quoted, that the evidence of cell development did not appear so satisfactory to him as it did to Mr. Solly and Mr. Birkett, and we have not so firm a faith in the revelations of the microscope, to induce us to dissent from what has always appeared to us, the most satisfactory account yet given of the character of this curious and rare disease, viz. that published by Mr. Curling, who describes it as *an extreme atrophy* of the bones, analogous to that occurring in a less degree in old age, the place of the osseous tissue being supplied by an excess of the natural fatty matter.

### XXIX. CASE OF FISTULOUS COMMUNICATION BETWEEN THE INTESTINUM ILEUM AND URINARY BLADDER, SIMULATING STONE IN THE BLADDER. By *W. C. Worthington*.

The patient was an old woman, aged 65, who, after an obscure abdominal affection, was seized with a disturbance of the urinary organs, attended with intense suffering. The leading symptoms were frequent and painful micturition, and bloody ropy and highly offensive urine, which was observed occasionally to deposit fragments of extraneous matter. On examination after death, the bladder was found to contain fæculent matter and portions of undigested food, such as currants, seeds, and other vegetable matter, which had escaped through an ulcerated opening in the fundus of the bladder communicating with the ileum. The author concludes by referring to a similar case observed by Camper.

